# Chemical Modulators for Targeting Autism Spectrum Disorders: From Bench to Clinic

**DOI:** 10.3390/molecules27165088

**Published:** 2022-08-10

**Authors:** Songhyun Lim, Sanghee Lee

**Affiliations:** 1 Creative Research Center for Brain Science, Brain Science Institute, Korea Institute of Science and Technology, Seoul 02792, Korea; 2 Department of HY-KIST Bio-Convergence, Hanyang University, Seoul 04763, Korea

**Keywords:** autism spectrum disorders, 5-hydroxytryptamine, glutamate, microbial metabolites, inflammatory cytokines

## Abstract

Autism spectrum disorders (ASD) are neurodevelopmental disorders characterized by diverse behavioral symptoms such as repetitive behaviors, social deficits, anxiety, hyperactivity, and irritability. Despite their increasing incidence, the specific pathological mechanisms of ASD are still unknown, and the degree and types of symptoms that vary from patient to patient make it difficult to develop drugs that target the core symptoms of ASD. Although various atypical antipsychotics and antidepressants have been applied to regulate ASD symptoms, these drugs can only alleviate the symptoms and do not target the major causes. Therefore, development of novel drugs targeting factors directly related to the onset of ASD is required. Among the various factors related to the onset of ASD, several chemical modulators to treat ASD, focused on serotonin (5-hydroxytryptamine, 5-HT) and glutamate receptors, microbial metabolites, and inflammatory cytokines, are explored in this study. In particular, we focus on the chemical drugs that have improved various aspects of ASD symptoms in animal models and in clinical trials for various ages of patients with ASD.

## 1. Introduction

Autism spectrum disorders (ASD) are a part of neurodevelopmental disorders and reveals a broad range of symptoms primarily characterized by repetitive behaviors, irritability, delayed development of language skills, and restricted social interactions [1,2,3,4]. It is known that abnormal brain developmental processes in ASD affect cognition, emotion, learning, and memory, thereby linking to abnormal behaviors [3]. Current study estimated that about 1 in 100 children around the world are diagnosed with ASD, and the prevalence is increasing over time [5]. According to the fifth edition of the Diagnostic and Statistical Manual of Mental Disorders from the American Psychiatric Association, ASD includes Asperger’s disorder and pervasive developmental disorder not otherwise specified (PPD-NOS) [4,6]. Despite the large number of patients, only limited cases of patients that have a recognized etiology and the pathological mechanism of ASD is still unknown in detail. [2,7]. Since there is no clear biomarker to objectively diagnose ASD, various diagnostic criteria and discrimination of the diversity of disorders are mainly based on monitoring behavioral features [2,8]. Moreover, ASD patients have a wide range of disabilities from a patient with a minimal effect on living to a patient where active support is needed. All these features of ASD such as ambiguous cause, no specific biomarkers, and broad range of patient spectrum make difficult to target core symptoms of ASD in drug discovery.

To find a therapeutic agent, an early drug discovery process accompanied by target identification and target validation, followed by assay development with specific biomarkers, leads to a drug development strategy [9]. However, the general early drug discovery process is very limited in ASD due to the lack of biomarkers and the unclear pathological mechanisms. Therefore, to date, there are no medications approved for use in treating the core symptoms of ASD [10]. Previously known drugs are primarily used to alleviate the behavioral symptoms of ASD, including irritability, repetitive behaviors, agitation, aggression, self-injury, anxiety, hyperactivity, insomnia, impulsivity, and inattention [3,10,11]. Several studies have reported the benefit of atypical antipsychotics including risperidone, aripiprazole, and lurasidone for behavioral improvement in certain symptoms of ASD such as aggression, hyperactivity, repetitive behavior, self-injury, irritability, and impulsivity [3,10,11,12,13,14,15,16,17,18,19,20,21,22,23]. Moreover, typical antipsychotics, antidepressants, and mood stabilizers are commonly used to regulate the behavioral symptoms of ASD in patients [10,11,14,15]. Along with various types of pharmaceuticals, behavioral interventions are also applied to control the symptoms of ASD [10,24,25,26].

Lack of exact pathological mechanisms, clinical targets, and specific biomarkers are considered characteristic features of ASD. These characteristics make it difficult to approach ASD from the perspective of chemical modulators. Moreover, drug repurposing with conventional drugs which have been developed to treat other neurological disorders validated their therapeutic effect for alleviating behavioral symptoms of ASD. Recently, the original targets of repurposing drugs have been found to have a function related to ASD. Therefore, in this review, we have summarized and discussed several types of chemical modulators by focusing on recent studies of drug candidates targeting ASD from their mode-of-action to proof-of concept (POC) in the preclinical stage and the therapeutic effects in clinical trials. We investigated a series of chemical modulators based on biochemical regulations including serotonin (5-hydroxytryptamine, 5-HT) and glutamate targeting core symptoms in ASD. For environmental regulations, the study of microbial metabolites targeting ASD was discussed. Finally, immune regulation associated with proinflammatory cytokines was investigated, as cytokine modulation could be a potential target for the treatment of ASD.

## 2. Pathophysiology of ASD

**Figure 1:**
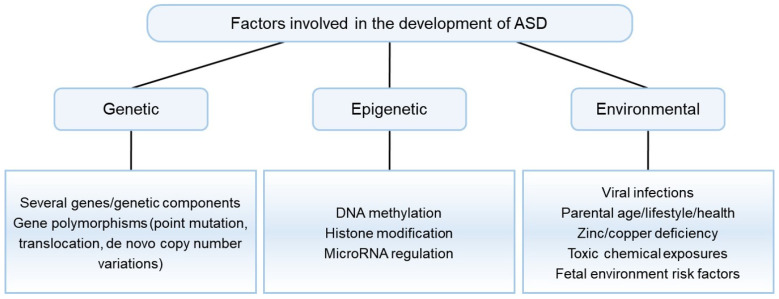
Factors involved in the development of autism spectrum disorder (ASD).

It is suggested that genetic, epigenetic, and environmental factors are involved in the process of the development and deepening of ASD (Figure 1). As various factors are involved in ASD, the genetic variation among individuals and the resulting variation of ASD symptoms appear widely. Although the exact pathological mechanism of ASD is unknown, genetic factors are strongly implicated to be involved in ASD pathophysiology. Sibling studies, twin studies, and the male-to-female ratio in ASD indicate the role of genetic components in the etiology of ASD [27,28,29,30,31]. Moreover, gene polymorphisms such as point mutation, translocation, and de novo copy number variations are possible risk factors in the development of ASD [3,32,33]. Several studies have mentioned the important role of de novo copy number variations in neurodevelopmental pathway of ASD [30,33,34,35,36,37].

Epigenetic modifications regulate chromatin structure and gene expression without the change in DNA sequence. Factors such as DNA methylation, histone modification, and microRNA regulate chromatin structure and gene expression, which plays a role in regulating brain development and can cause neurodevelopmental disorders including ASD [33].

Environmental factors, mostly in the prenatal period, are also an important cause of ASD. Viral infections, parental age and health, zinc and copper deficiency, toxic chemical exposures, various fetal environments, and perinatal/natal risk factors are involved [38,39]. Along with genetic and environmental factors, various central neurotransmitter systems involved in initial brain development, serotonin (5-hydroxytryptamine, 5-HT), gamma-aminobutyric acid (GABA), dopamine, glutamate, histamine, and acetylcholine are suggested to play a significant role in onset and development of ASD [3,32].

## 3. Classification of Chemical Modulators

### 3.1. Neurotransmitters

#### 3.1.1. 5-HT Receptors (5-HTRs)

Among various neurotransmitters, 5-HT is especially important in brain development and ASD symptoms including aggression, anxiety, insomnia, and social behavior as it regulates cell division, differentiation, and synaptogenesis [40,41]. Elevated 5-HT levels in plasma and platelets (hyperserotonemia) have been detected in more than 25% of children with autism at the early stage of ASD development, which is often observed in people with developmental disorders [3,41,42]. Moreover, changes in 5-HT synthesis and 5-HTR density with age are highly related to ASD development and progress [40]. Several studies have indicated that targeting 5-HTRs could be effective in treating the core symptoms of ASD [43].

Various types of compounds including atypical antipsychotic drugs, antidepressants, and selective serotonin reuptake inhibitors (SSRIs) have been applied to target seven families of 5-HTRs in ASD treatments (Table 1). First, several types of atypical antipsychotic drugs were prescribed for ASD patients in clinical settings. Aripiprazole, the United States Food and Drug Administration (FDA)-approved antipsychotic, was demonstrated as both an agonist and antagonist for several 5-HTR types and has been used to alleviate irritability in ASD [3,16,44,45,46]. Comparably, other pharmaceuticals such as risperidone and lurasidone showed antagonistic activity against various types of 5-HTR as well as dopamine receptors, and these drugs were effective against irritability, aggression, temper outburst, and the self-injurious behavior of children with autism [2,3,47,48,49,50,51]. Antidepressant agents targeting the 5-HT level have been used to manage not only anxiety and obsessive–compulsive symptoms but also other complex autistic symptoms. Vortioxetine, another antidepressant, 5-HT transporter, and 5-HT_1A_/5-HT_1B_ activator, reduced repetitive behavior but showed little effect on enhancing sociability in the rodent model of ASD [52]. Clomipramine, a tricyclic antidepressant and a potent SSRI, has been FDA-approved for treating patients with obsessive–compulsive disorder, which is strongly related to ASD symptoms [11,14]. Clomipramine was effective in reducing abnormal social interaction, compulsive symptoms, and stereotypic ASD behaviors in the treated group compared to the placebo group in a double-blind study [53]. Other SSRIs, fluoxetine, fluvoxamine, and sertraline, showed an improvement of symptoms associated with ASD including repetitive behavior, aggression, language usage, abnormal social interactions, cognition, anxiety, irritability, and agitation [54,55,56,57,58,59]. All these applications of known drugs indicated 5-HTR engagement to alleviate ASD symptoms and to address the unmet needs for the specific regulation of 5-HTR. For this purpose, several studies for the development of a specific chemical modulator for 5-HTR in ASD treatment have been reported. (+)-5-FPT, a 5-HT_1A_R/5-HT_2C_R agonist and 5-HT_7_R antagonist, was developed and studied in a mouse model to reduce repetitive behavior through 5-HT_1A_R agonism and audiogenic seizure through 5-HT_2C_R agonism [60,61]. The administration of 8-OH DPAT, a 5-HT_1A_R/5-HT_7_R agonist, to a valproate-induced rat ASD model improved social interaction and reduced anxiety and hyperactivity [62]. A recent study designed and synthesized a compound 2c, which acts as an antagonist against 5-HT_7_R [63]. In a transgenic mouse model of ASD, 2c reduced the level of repetitive behaviors to a level similar to the wildtype mice. These results demonstrated that investigation of a 5-HTR modulator could potentially lead to the development of effective drugs for ASD stereotypes.

#### 3.1.2. Glutamate Receptors (GluRs)

Glutamate, the primary excitatory neurotransmitter in the brain, plays a role in neuronal developmental processes such as learning and memory formation [2,64,65]. The disruption of the glutamate system is involved in ASD development, because ASD may be caused by a disruption of the balance between exhibition and inhibition in the brain [2]. An increased glutamate level in the blood and platelets of individuals with autism has been reported [65,66,67,68]. This evidence suggests that abnormalities in the glutamatergic signaling pathways occur in ASD [65]. A previous study identified that in a valproic acid (VPA)-induced ASD rat model, a substantial increase in the expression of genes encoding subunits of the ionotropic GluRs was detected [69]. Therefore, GluRs could be a plausible target for treatment of ASD symptoms, and various preclinical and clinical cases have been reported (Table 2).

There are two types of GluRs, ionotropic and metabotropic, and three subtypes of ionotropic glutamate receptors including kainite, α-amino-3-hydroxy-5-methyl-4-isoxazolepropionic acid (AMPA), and N-methyl-D-aspartate (NMDA) [2,64]. Glutamate ionotropic receptor kainite type subunit 2 (GRIK2), which encodes for GluR6, is a strong candidate gene for ASD treatment [2,65,70,71,72]. GRIK2 is localized in the autism-specific region on chromosome 6q21, and its encoded protein GluR6 is involved with ASD symptoms such as anxiety, learning, and memory [70,71]. Unfortunately, to date, there is no preclinical drug development targeting abnormal GluR6 activity to treat ASD. Meanwhile, previous studies reported that modulation of AMPA reduced social impairments in animal models of ASD [73,74]. In particular, ampakine Cx546, an AMPA receptor positive allosteric modulator, improved social interaction in genetic mouse models of ASD [75]. These results indicate that the mechanism for enhancement of excitatory synaptic transmission would be a novel candidate to treat ASD symptoms.

Another therapeutic target in ASD is the glutamate hyperfunction at NMDA receptors [2,70]. A representative NMDA antagonist, memantine, has been widely studied for treatment of people with ASD symptoms. An open-label add-on therapy of memantine offered to patients with ASD or PPD-NOS over a 21-month period showed improvements in language function, social behavior, and self-stimulatory behaviors [76]. Another study of a randomized single-blind clinical trial for children under 14 with ASD introduced memantine as a new adjunct drug with applied behavior analysis (ABA), which resulted in the improvement of ASD symptoms, compared to the ABA-only group [77]. As well as children, an open-label trial of memantine for adults with ASD showed a significant improvement in ADHD and anxiety after a 12-week intervention [78]. A randomized controlled trial of memantine for the treatment of social impairment in adolescents with ASD is currently underway (ClinicalTrials.gov: NCT01972074). D-cycloserine, a partial agonist at NMDA receptors, has shown positive effects on the social behavior of ASD in animal models [79,80]. Based on the therapeutic effects of D-cycloserine on the ASD animal models, several clinical trials were conducted to validate a reduction in the social deficit in people with ASD. A single-blind placebo lead-in phase study for 2 weeks to examine the effects of D-cycloserine on age 5 and older subjects showed a significant improvement in social impairment in ASD [81]. A double-blind randomized 10-week trial on older adolescents and young adults with ASD showed that D-cycloserine caused a clinically and statistically significant improvement in social deficits, a major impairment in ASD [82]. A 10-week double-blind placebo-controlled trial on children ages 5–11 years showed an adjunctive effect on increasing the sustained benefit from short-term social skills intervention [83].

Metabotropic GluRs are expressed at presynaptic sites and glial cells, thereby regulating neurotransmitter release, and play critical roles in neuroprotection, glial-neuron communication, and glutamate release, implicating their regulation in neuronal development and neurological disorders [84,85]. Various agonists or antagonists of metabotropic GluR subtypes have been considered as alleviators of ASD symptoms. A previous study investigated the potential effect of the metabotropic GluR5 antagonist, fenobam, and the GluR1 antagonist, JNJ16259685 [86]. Deletion of the eukaryotic initiation factor 4E-binding protein 2 (4E-BP2) gene in mouse showed an imbalance in excitatory and inhibitory neurotransmission and ASD-like behaviors. Treatment of fenobam and JNJ16259685 in 4E-BP2-deleted mice led to a reduction in repetitive behaviors and improved social behaviors. Moreover, 2-methyl-6-phenylethynyl-pyridine (MPEP), an antagonist of the metabotropic GluR5, reduced the repetitive behavior of various ASD mouse models in previous studies [87,88]. Acamprosate, a weak NMDA receptor antagonist and also a metabotropic GluR5 antagonist, was administered to children with ASD for an 8-week open-label trial [89]. Although the scale of the trial was relatively small, the results showed positive effects of acamprosate on verbalization, attention, social behavior, and hyperactivity. As for current trial results to look forward to, a phase 1 double-blind placebo-controlled study of acamprosate in ASD is ongoing (ClinicalTrials.gov: NCT01813318).

**Table 2:** Glutamate-related pharmacological agents and their potential effects on treating ASD symptoms. AMPA: α-amino-3-hydroxy-5-methyl-4-isoxazolepropionic acid, NMDA: N-methyl-D-aspartate, GluR: Glutamate receptor, MPEP: 2-methyl-6-phenylethynyl-pyridine.

Compounds	Structures	Targets	Stage	Effects
Cx546	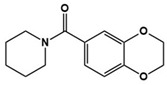	AMPA receptor positive allosteric modulator	Preclinical	Improved social interaction [75]
Memantine/Memantine hydrochloride (Namenda)	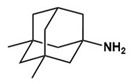	NMDA antagonist	Clinical	Improvements in language function, social behavior, ADHD, anxiety and self-stimulatory behaviors [76,77,78]
D-cycloserine	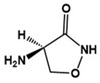	Partial agonist at NMDA receptors	Preclinical	Positive effects on social behavior [79,80]
			Clinical	Positive effects on social behavior [81,82], increasing the sustained benefit from short-term social skills intervention [83]
Fenobam	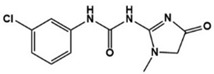	Metabotropic GluR5 antagonist	Preclinical	Reduction of repetitive behaviors, improved social behaviors [86]
JNJ16259685	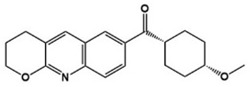	GluR1 antagonist	Preclinical	Reduction of repetitive behaviors, improved social behaviors [86]
MPEP	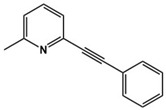	Metabotropic GluR5 antagonist	Preclinical	Improved repetitive behavior [87,88]
Acamprosate	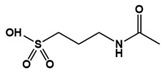	A weak NMDA receptor antagonist, metabotropic GluR5 antagonist	Clinical	Positive effects on verbalization, attention, social behavior and hyperactivity [89]

### 3.2. Microbial Metabolites

**Figure 2:**
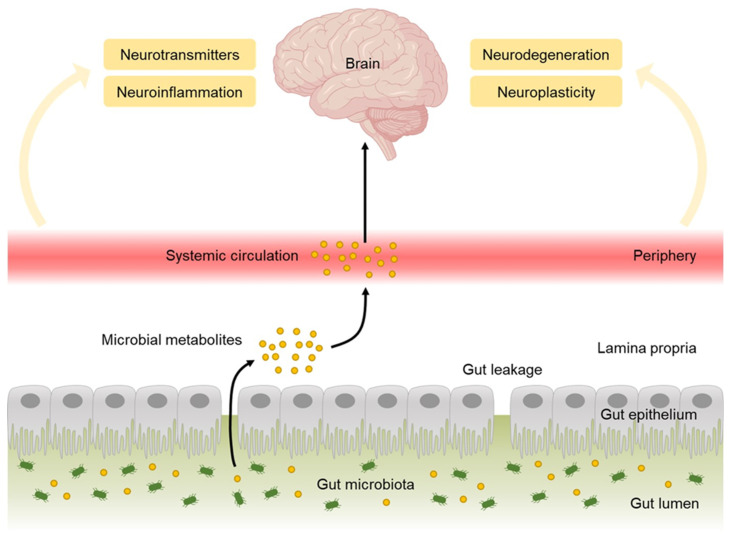
Microbiota gut–brain axis mechanisms focused on microbial metabolites and their influences on the brain. (Brain image from BioRender.com).

**Figure 3:**
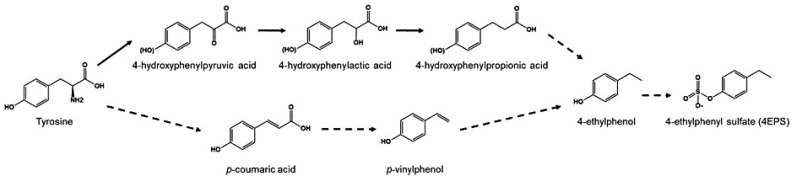
Formation of 4-ethylphenyl sulfate (4EPS) from tyrosine. Solid arrows show established reactions, and dotted arrows represent expected reactions.

Recent studies have uncovered the important role of the gut–brain axis and microbiota in brain physiology–pathology and various neurological disorders (Figure 2) [90,91]. One of the environmental factors underlying the etiology of ASD is viral infection and consequent changes in the gut microbiome [92]. The result of reinducing or treating gut microbial ecology in various mouse models with ASD-like symptoms showed improved social behavior [92,93,94]. Furthermore, the regulation of neuroactive microbial metabolites and crosstalk with the immune system are attracting growing attention as a therapeutic approach for ASD. This approach avoids crossing the blood–brain barrier in drug discovery. Recently, an open-label phase 1b/2a trial was conducted to remove increased levels of toxic microbial metabolites, which are related to ASD-associated behaviors [8]. 4-ethylphenyl sulfate (4EPS), one of the gut microbial metabolites, is eventually produced by 4-ethylphenol through various intermediates starting from tyrosine, a precursor of various mammalian neurotransmitters (Figure 3) [95]. 4EPS is initially produced in gut microbiota and then circulates through the whole body by blood. 4EPS is highly observed in the plasma of individuals with ASD symptoms [95,96]. AB-2004, also known as AST-120, is an oral gastrointestinal-restricted adsorbent, which has an affinity for small aromatic/phenolic molecules, especially uremic toxins including those of gut bacteriological origin. In other words, AB-2004 works as a toxin scavenger generated in the microbiome. Treatment of AB-2004 reduced the level of 4EPS in mice and reduced anxiety and behavioral deficits [8]. Furthermore, the administration of AB-2004 for an adolescent ASD population in a phase 1 clinical trial for 8 weeks confirmed the decreased level of gut microbiological metabolites in plasma and urine, improved gastrointestinal health, and decreased symptoms of anxiety and irritability (ClinicalTrials.gov: NCT04895215).

### 3.3. Inflammatory Cytokines

The central nervous system is closely correlated with the immune system. Neuropeptides, which are formed by neurons, act as chemoattractants to recruit immune cells, and various effects of immune factors on neuronal migration and neurochemical receptors are related to the genetic risk of ASD [97,98]. In particular, the abnormalities of the immune system and consequent generation of autoantibodies and cytokine alternations/imbalance are closely connected with the ASD phenotype [99,100,101]. The formation of maternal autoantibodies is one of the most important aspects of ASD symptomatology in the prenatal period. Indirect evidence shows that the parents of a child with ASD, especially mothers, have a higher incidence of autoimmune disease [97]. In addition, abnormal brain enlargement in children with ASD was only observed in the case where the mother had the maternal autoantibodies for fetal brain protein [102].

The risk of developing ASD symptoms in children increased when the mothers were exposed to maternal inflammation during pregnancy [103,104,105]. A maternal immune activation (MIA) model was generated by administration of a viral mimetic polyinosinic-polycytidylic acid (poly(I:C)) to activate the maternal immune system of pregnant mice, and the MIA-induced offspring showed representative symptoms of ASD such as impaired sociability and reactive behavior [106]. Over the last half-decade, subsequent experimental evidence has revealed that maternal inflammation and the subsequent changes in gut microbiota affect neurobehavior in the MIA model, and these results suggest the significant role of MIA in ASD [93,107,108].

In line with the outcome of increased ASD risk in offspring by MIA, maternal infection leads to maternal proinflammatory cytokine release and Th17 cell activation, which can heighten the risk for ASD in offspring, combined with the fetus’ immune state and genetic predisposition [109]. When MIA was induced in two types of mice with different microbiota compositions, ASD symptoms were observed only in the mice with higher interleukin (IL)-17a production [110]. The result proved the significant role of IL-17a regulated by the prenatal microbiota composition, which closely relates to the onset of ASD by affecting the immune profiles.

As well as IL-17a, various cytokines play an important role in neurodevelopment by modulating brain function and neural activity [111]. For example, proinflammatory cytokines such as tumor necrosis factor α (TNFα), IL-6, and IL-1β are known to cross the blood–brain barrier and affect the hypothalamus [97,112]. As one of the effects of the immune system in the prenatal period, elevated levels of IL-1β and IL-4 are associated with childhood ASD development [101,113]. Cytokines are also closely related to the pathogenesis and maintenance of ASD in the postnatal period. A previous study reported that the plasma levels of IL-1β, IL-1 receptor antagonist, IL-5, IL-8, IL-12(p70), IL-13, IL-17, and growth-related oncogene-α in male children with ASD were elevated compared to healthy male subjects [114]. Moreover, several studies have confirmed the elevated level of cytokines in ASD patients in various age ranges [111,115,116,117,118,119,120]. Thus, it is considered that an altered profile of cytokines or cytokine overproduction is involved in ASD pathophysiology.

Recently, new treatments by immunomodulation have been introduced through several studies along with mounting evidence of the link between the immune response and ASD pathophysiology (Table 3). In particular, increased proinflammatory cytokines along with JAK (Janus tyrosine kinase)/STAT (signal transducer and activator of transcription) signaling have been identified in children with ASD; therefore, targeting JAK/STAT signaling has therapeutic potential for ASD. Luteolin, known as an antioxidant and a citrus bioflavonoid, is an inhibitor of neuronal IL-6-induced JAK2/STAT3 phosphorylation [4,121]. A case series administering NeuroProtek, which mainly contains luteolin, to children with ASD aged 4–14 years was published [122]. The study reported significant improvement in eye contact and attention to directions in about half the patients and social interactions in 30–50% of patients. Another open-label pilot study for children with ASD provided them with a luteolin formulation for 26 weeks. The results showed significant improvement in adaptive functioning and overall ASD behavior with no major adverse effects [123]. Diosmin, a structural analog of luteolin, is also an inhibitor of the JAK2/STAT3 signaling pathway. A study that orally administered diosmin in a MIA mouse model showed significantly reduced abnormal behavior and neuropathological abnormalities in the offspring [121]. Tyrphostin AG126, a protein tyrosine kinase inhibitor, is known to play a role in neuroprotection and neuroinflammation [124,125]. The effects of tyrphostin AG126 have been demonstrated in two animal studies using a BTBR mouse model of ASD. Treatment of tyrphostin AG126 resulted in an improvement of repetitive and social behavior, as well as the downregulation of IL-21/IL-21R and IL-17A cytokine levels and the JAK/STAT pathway [126,127]. Resveratrol is another drug attenuating proinflammatory cytokines such as IL-17A, IL-6, interferon (IFN)-γ, and TNF-α and activation of the JAK1/STAT3 pathway [4,128]. Several studies have demonstrated the behavioral effects of resveratrol using animal models of ASD. Core ASD symptoms such as repetitive behavior and social deficits were prevented by resveratrol [129,130,131,132,133]. Based on the positive results for ASD in animal studies, a double-blind and placebo-controlled randomized trial was conducted with children with ASD [134]. Although there was no significant improvement in the primary outcome, add-on pharmacotherapy with resveratrol showed an improvement in hyperactivity at the end of the study.

In addition, several compounds that inhibit proinflammatory cytokines have been applied to clinical trials for children with ASD. Celecoxib, an inhibitor against cyclooxygenase-2, has been reported as significantly effective for ASD symptoms [135]. A randomized double-blind placebo-controlled trial, which used celecoxib adjunctively with risperidone, demonstrated positive effects on social deficit, stereotyped activity, and irritability in children with ASD [136]. Another proinflammatory cytokine inhibitor, pentoxifylline, was also applied as an adjunctive treatment to risperidone to control the behavioral aspects of ASD [137]. A double-blind placebo-controlled clinical trial with children diagnosed with ASD demonstrated improvements in social withdrawal/lethargy, stereotyped behavior, irritability, hyperactivity/noncompliance, and inappropriate speech.

The normalization of increased IL-6 by purinoceptor suggested a therapeutic effect on ASD. Suramin is a P2-purinoceptor antagonist [138]. A recent study demonstrated that blocking the purinergic receptor with suramin increased the survival of postnatal neural progenitor cells [139]. Based on the significant role of neural progenitor cells in brain development, the effect of suramin was addressed in various mouse models of ASD. A study with a VPA-induced ASD mouse model showed restored sociability and decreased anxiety after treatment with suramin [140]. Moreover, suramin induced the normalization of an elevated level of IL-6 in ASD mice. In other studies, using MIA mouse models exposed to poly(I:C), suramin resulted in reversed social abnormalities [141,142]. A double-blind placebo-controlled randomized clinical trial was conducted with ten male subjects with ASD ages 5–14 years. A single intravenous treatment of suramin led to improvements in social interaction and language and reduced repetitive/restricted behaviors [143].

**Table 3:** Immune regulation agents and their potential effects on treating ASD symptoms. IL: interleukin, JAK/STAT: janus tyrosine kinase/signal transducer and activator of transcription, IFN: interferon, TNF: tumor necrosis factor.

Compounds	Structures	Targets	Stage	Effects
Luteolin	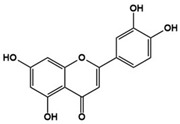	Inhibitor of neuronal IL-6-induced JAK3/STAT3 phosphorylation	Clinical	Improved eye contact, attention to directions, social interactions [122], improvement in adaptive functioning and overall ASD behavior [123]
Diosmin	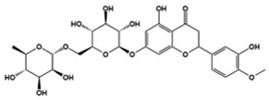	Inhibitor of neuronal IL-6-induced JAK3/STAT3 phosphorylation	Preclinical	Opposed abnormal behavior and neuropathological abnormalities [121]
Tyrphostin AG126	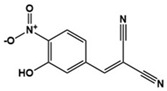	Protein tyrosine kinase inhibitor, IL-21/IL-21R, IL-17A, JAK/STAT downregulator	Preclinical	Improvement of repetitive and social behavior [126,127]
Resveratrol	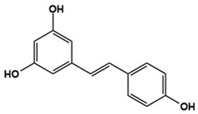	IL-17A, IL-6, IFN-γ, TNF-α, and JAK1/STAT3 downregulator	Preclinical	Improvement in repetitive behavior, social deficits [129,130,131,132,133]
			Clinical	Improvement in hyperactivity [134]
Celecoxib	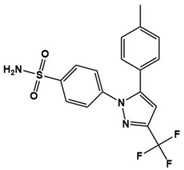	Cyclooxygenase-2 inhibitor	Clinical	Improvement in social deficit, stereotyped activity, and irritability [136]
Pentoxifylline	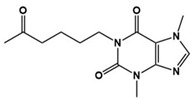	Pro-inflammatory cytokine inhibitor	Clinical	Improved social withdrawal/lethargy, stereotyped behavior, irritability, hyperactivity/noncompliance, and inappropriate speech [137]
Suramin	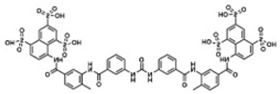	P_2_-purinoceptor antagonist, leads to IL-6 decrease	Preclinical	Improvement in sociability [140,141,142] and anxiety [140]
			Clinical	Improvement in sociability, language, and repetitive/restricted behaviors [143]

## 4. Challenges and Future Perspectives

The challenging part of developing chemical modulators to cure ASD is that the pathological mechanism of ASD is not clear and the degree of symptoms varies greatly from patient to patient. The most challenging part is the lack of a therapeutic target for ASD. Nevertheless, the continuous effort to elucidate biological targets for ASD has progressed. The strategies discussed in this study, such as regulation for neurotransmitters, microbial metabolites, and pro-inflammatory cytokines, have been elucidated based on clinically observed features in ASD patients.

Successful drug development for ASD requires the development of appropriate biomarkers and animal models that target the core symptoms of ASD, and POC for alleviating ASD symptoms must be validated in connection with clinical evidence. Based on the validated POC, it is also necessary to expand potential seed compounds. For these purposes, various immunomodulators for regulating cytokines or microbiome metabolites would be a novel approach for the identification of potential candidates.

## 5. Conclusions

In this review, we focused on chemical modulators, which can be potential drugs for the treatment of ASD. We especially focused on pharmacological agents that are known to target 5-HT and glutamate receptors, microbial metabolites, and inflammatory cytokines. Based on the analysis of 50 publications and clinical trial information on ClinicalTrials.gov, we investigated the therapeutic effect of 25 chemical modulators for ASD.

Antipsychotics and antidepressants once applied to several neurological diseases have been reported as agonists or antagonists to various types of 5-HTR. Moreover, other compounds such as (+)-5-FPT, 8-OH DPAT, and 2c act as agonists or antagonists to the 5-HTR family, especially to 5-HT7R. These drugs have been effective for a reduction in repetitive behavior and the improvement in social behavior and other symptoms related to ASD. In addition, the effects of the selective serotonin reuptake inhibitor on ASD symptoms fully demonstrated that 5-HT is a potential target for the treatment of ASD. Moreover, there are two types of receptors for glutamate, ionotropic and metabotropic. Drugs acting as an allosteric modulator, antagonist, or agonist for two types of glutamate receptors showed positive results for ASD symptoms. In particular, memantine, D-cycloserine, and acamprosate, which are agonists/antagonists for the NMDA receptor, a type of ionotropic receptor, showed a significant improvement in the behavioral symptoms of ASD in clinical trials for patients of various ages. Microbial metabolites and inflammatory cytokine levels are environmental factors that are known to be associated with the expression of ASD symptoms. AB-2004, a drug that removes 4EPS, one of the toxic microbial metabolites, showed alleviation of anxiety and other behavioral deficits in an ASD mouse model and a subsequent clinical trial. This suggests the effect of the gut–brain axis system centered on the systemic circulation of gut microbial metabolites on the occurrence and deterioration of ASD. Meanwhile, an MIA model has been widely used as an experimental ASD model based on the fact that maternal infection and proinflammatory cytokines increase the risk of ASD development in offspring. In conjunction with these characteristics, inhibitors/downregulators for proinflammatory cytokines such as IL-6 and IL-17 and subsequently activated pathways were applied to patients with ASD symptoms. In particular, tyrphostin AG126, resveratrol, and suramin showed positive effects on reducing repetitive behavior and improving social interaction, core symptoms of ASD. Overall, various chemical modulators are being developed for the treatment of the core symptoms of ASD focused on neurotransmitters, harmful microbial metabolites, proinflammatory cytokines, and immune system regulation.

## Figures and Tables

**Table 1:** 5-HT-related pharmacological agents and their potential effects on treating ASD symptoms. 5-HT: serotonin (5-hydroxytryptamine), 5-HTRs: 5-HT receptors. SSRI: selective serotonin reuptake inhibitor.

Compounds	Structures	Targets	Stage	Effects
Aripiprazole	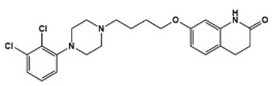	Partial 5-HT_1A_R/5-HT_2C_R, agonist, 5-HT_1B_R/5-HT_1D_R/5-HT_2A_R/5-HT_2C_R/5-HT_3A_R/5-HT_6_R/5-HT_7_R antagonist	Clinical	Alleviating irritability [16,44,46]
Risperidone	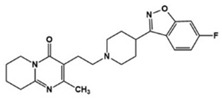	5-HT_1A_R/5-HT_1D_R/5-HT_2A_R/5-HT_2C_R/5-HT_7_R antagonist	Clinical	Effective in irritability, aggression, temper-outburst and self-injurious behavior [47,48,49]
Lurasidone	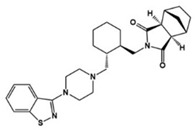	5-HT_2A_R/5-HT_7_R antagonist, 5-HT_1A_R partial agonist	Clinical	Alleviating irritability [51]
Clomipramine	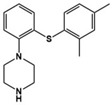	Potent SSRI	Clinical	Alleviating obsessive-compulsive disorder and abnormal social interaction [53]
Vortioxetine	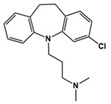	5-HT transporter, 5-HT_1A_/5-HT_1B_ activator	Preclinical	Reduced repetitive behavior [52]
Fluoxetine	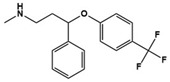	SSRI	Clinical	Improvement in repetitive behavior, social interactions, language and cognition [54,56]
Fluvoxamine	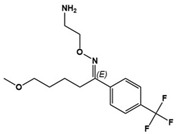	SSRI	Clinical	Improvement in repetitive behavior, maladaptive behavior, aggression, social interaction and language usage [55,59]
Sertraline	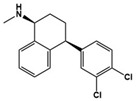	SSRI	Clinical	Improvement in repetitive behavior, aggression, anxiety, irritability and agitation [57,58]
(+)-5-FPT	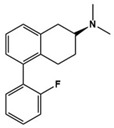	5-HT_1A_R/5-HT_2C_R agonist, 5-HT_7_R antagonist	Preclinical	Reduced repetitive behavior [60,61]
8-OH DPAT	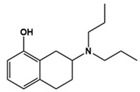	5-HT_1A_R/5-HT_7_R agonist	Preclinical	Improvement in social interaction, anxiety and hyperactivity [62]
2c	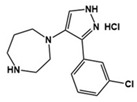	5-HT_7_R antagonist	Preclinical	Reduced repetitive behavior [63]

## Data Availability

Not applicable.
